# Integrating digital health technologies to advance personalized cancer rehabilitation

**DOI:** 10.1097/JS9.0000000000003207

**Published:** 2025-08-07

**Authors:** Jie Wang, Yizhen Wang, Liqiong Tian

**Affiliations:** Department of Rehabilitation Medicine, Changde Hospital, Xiangya School of Medicine, Central south University (The first people’s hospital of Changde city), Changde, Hunan, China

*Dear Editor,*

We read with great interest the comprehensive review by Chen *et al* titled “A novel approach to cancer rehabilitation: assessing the influence of exercise intervention on postoperative recovery and survival rates”[1]. The authors provide a valuable synthesis of evidence demonstrating how structured exercise interventions – including aerobic, resistance, and flexibility training – significantly enhance functional recovery, reduce complications, and potentially improve survival outcomes across multiple cancer types. We commend the authors for highlighting the critical need for personalized rehabilitation protocols and identifying research gaps in long-term effectiveness across diverse populations.

To extend this important work, we propose the integration of digital health technologies as a transformative approach to address existing challenges in scalability, personalization, and long-term adherence. Artificial intelligence (AI)-driven platforms can optimize exercise prescription by analyzing multimodal data (e.g., electronic health records, wearable sensors, patient-reported outcomes) to dynamically adapt interventions based on individual tolerance, comorbidities, and treatment phase. For instance, AI-driven clinical decision support systems are reshaping how cardiotoxicity is managed in real time. By integrating patient similarity algorithms and ML-based decision models, AI facilitates personalized treatment modifications and exercise-based prevention strategies[2]. Reinforcement learning (RL) represents a compelling method within ML for tailoring and adapting exercise programs to individual users. In RL, a decision-making agent performs actions that lead to preferred states within its environment[3].

Wearable devices and remote monitoring tools offer unprecedented opportunities to enhance the implementation framework discussed by Chen *et al*. Wearable device use is an innovative and effective way to promote physical activity and improve health-related outcomes in breast cancer survivors[4].Cancer survivors show an increase in physical activity when using consumer wearable activity trackers. Increased physical activity plays an important role in alleviating many adverse effects of breast cancer therapy as well as improving morbidity and mortality[5].This aligns with the authors’ emphasis on preoperative conditioning and postoperative recovery, where continuous data streams could refine ERAS (Enhanced Recovery After Surgery) protocols. Moreover, many of the newer activity monitors capture data beyond physical activity (e.g., heart rate, sleep, sedentary time) and can be synced with companion web-based or mobile apps that offer additional tools to track and offer supplementary feedback on related lifestyle behaviors (e.g., diet, sleep, stress), provide health education, and model/demonstrate target behaviors[6].

Furthermore, tele-rehabilitation platforms incorporating virtual reality (VR) and augmented reality could overcome geographic and resource barriers noted in the review. The virtual environment provided stimuli and pleasure, encouraging participants to be more active. On the other hand, VR can directly affect exercise rehabilitation through interaction. The interaction associated with VR generates visual stimuli through which patients can identify differences between their movements and the correct ones[7]. These technologies align with the biological mechanisms explored by Chen *et al*, as real-time form correction may optimize immune modulation through precise dosing of exercise intensity.

Future research should prioritize integrating these digital solutions within randomized trials measuring long-term survival and recurrence endpoints. Collaborative frameworks like the American College of Sports Medicine’s Moving Through Cancer initiative provide ideal platforms for evaluating technology-augmented rehabilitation[8]. By leveraging digital tools to operationalize the excellent foundational work presented by Chen *et al*, we can accelerate the translation of evidence-based exercise oncology into scalable, precision rehabilitation models. (We ensure this article is compliant with the TITAN Guidelines[9].)

## Data Availability

Not applicable.
